# Supplementary material for: Unraveling mitotic protein networks by 3D multiplexed epitope drug screening
Source: Mol Syst Biol. 2018 Aug 13;14(8):e8238. doi: 10.15252/msb.20188238 (PMC6088390; doi:10.15252/msb.20188238)
Supplement: Supplementary file 1 — Appendix [file MSB-14-e8238-s001.pdf]

Appendix for:

Unraveling mitotic protein networks by

3D multiplexed epitope drug screening

Lorenz Maier[1,2,3,+], Stefan Kallenberger[1,2,3,+], Katharina Jechow [2], Marcel Waschow[2], Roland Eils [1,2,3]*, Christian Conrad [1,2]*

Table of contents

**Appendix Figure S1 - Cell volumes, abundance and concentration changes.**	2
**Appendix Figure S2 - Predicted localization affinities.**	4
**Appendix Figure S3 - Importance of affinity parameters for model fit.**	6
**Appendix Figure S4 - Average mutual affinities of all inhibitor effects.**	7
**Appendix Figure S5 – Simple affinity model for two species.**	7
**Appendix Figure S6 – Sequential forward selection of mutual affinities.**	8
**Appendix Figure S7 - Results of multi-start local optimizations.**	8
**Appendix Table S1 - Assay proteins, inhibitors and cell lines.**	9

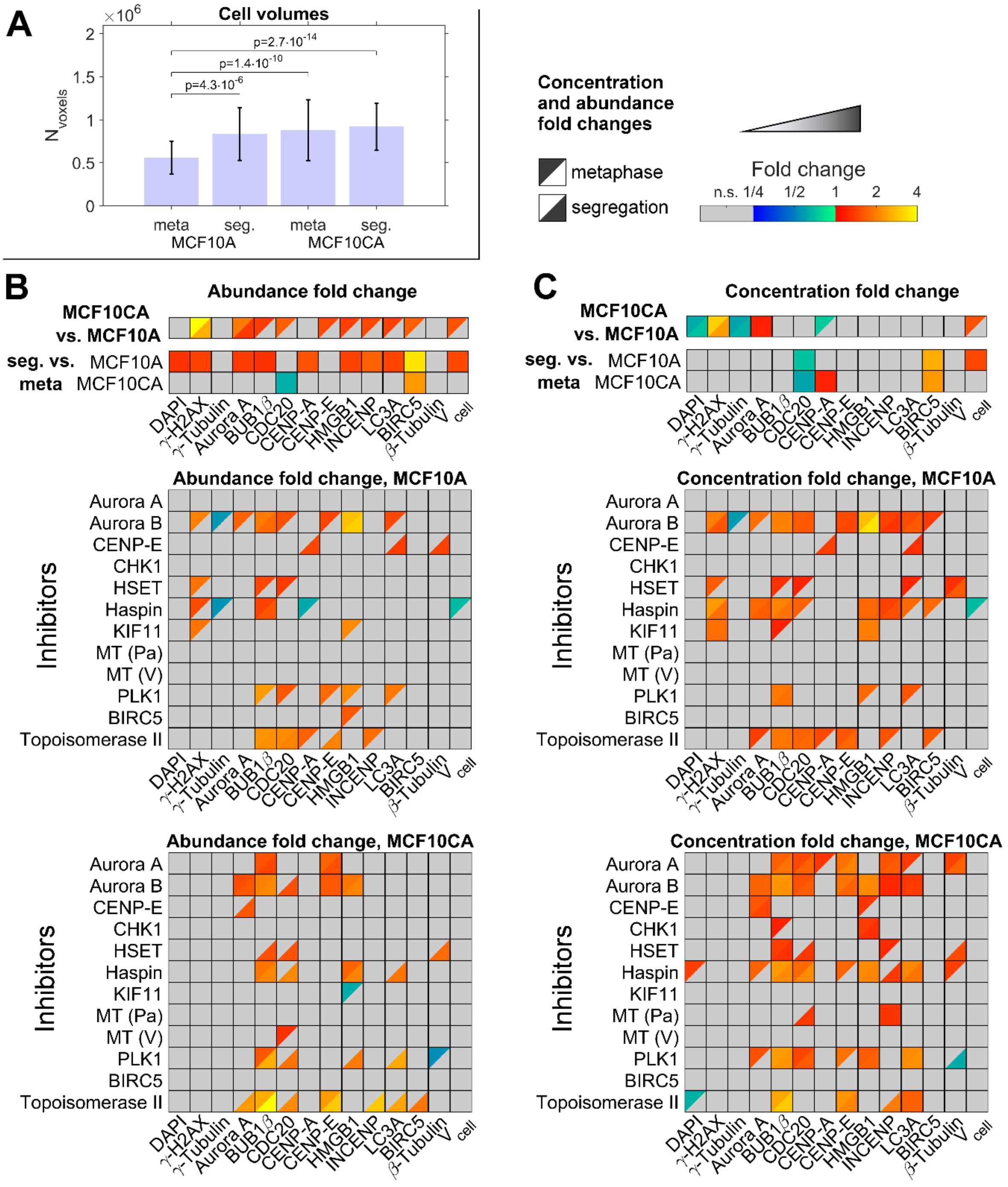

Appendix Figure S1 - Fold changes in measures of abundance or concentration in single cells.

A Cell volumes of MCF10A and MCF10CA cells during metaphase or segregation. For significant comparisons, p-values are indicated (Welch's t-test followed by Bonferroni correction for 6 tests, $p<0.05/6$; error bars: standard deviations; seg., segregation).

B Fold changes in measures of abundance related to the cell line (top panel, MCF10CA vs. MCF10A), mitotic phase (segregation vs. metaphase) or inhibitor treatments. Effects related

to cell line or inhibitors were indicated separately for metaphase (upper left triangles) and segregation (lower right triangles). All significant effects were visualized (Welch’s t-tests performed for 18 ROIs followed by Bonferroni correction for 52 tests in each measured species, $p<0.05/52$). Only Haspin had slight effects on volume estimates during metaphase. For all other inhibitors, effects on abundance measures were similar to effects on concentration measures. Effects related to abundance measures were less prominent than for concentration measures. This can be explained as a result of multiplying intensities with volume estimates to obtain abundance measures, which increases the influence of cell volume estimates as an error source (MT, microtubule inhibitor; Pa, paclitaxel; V, vinblastine; n. s., not significant).

C Fold changes in measures of concentration related to the cell line (top panel, MCF10CA vs. MCF10A), mitotic phase (segregation vs. metaphase) or inhibitor treatments as in B (see also Figs 3A and B).

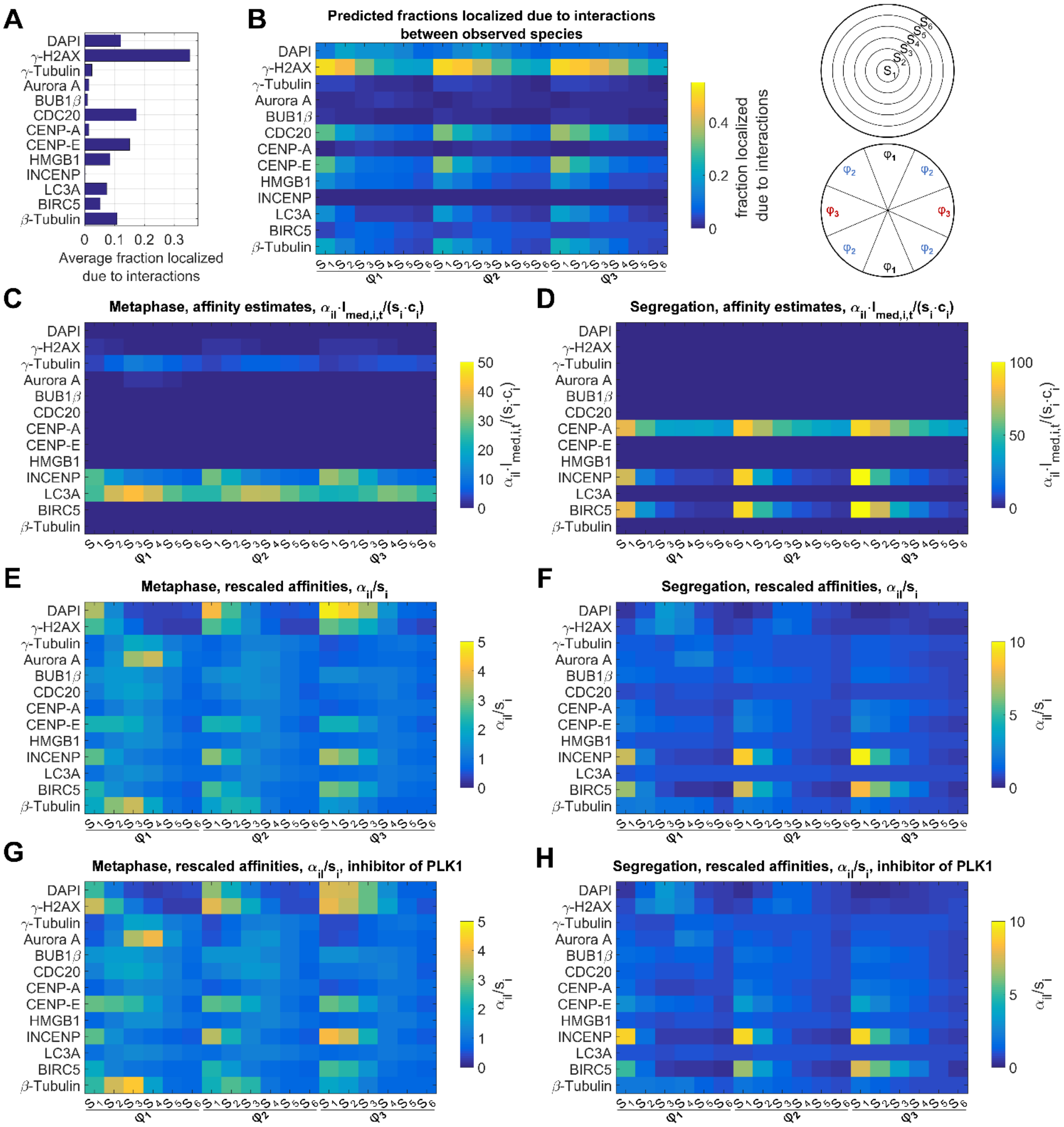

Appendix Figure S2 - Predicted localization affinities.

A Predicted fractions of proteins recruited to mitotic ROIs due to mutual affinities between proteins.

B Predicted fractions of proteins recruited due to mutual affinities between proteins, distinguished by eccentricity intervals (S_1 to S_6) and orientations (φ_1 to φ_3) as indicated by schematic maps.

C, D Affinity estimates $\tilde{\alpha}_{il} = \alpha_{il} I_{med,i,t} / (s_i c_i)$, before dividing by scaling factor estimates, during metaphase or segregation obtained by model fitting to dataset from untreated cells (see Appendix Supplementary Methods for details).

E, F Rescaled untreated localization affinities α_{il} / s_i during metaphase or segregation.

G, H Rescaled localization affinities α_{il} / s_i upon treatment with PLK1.

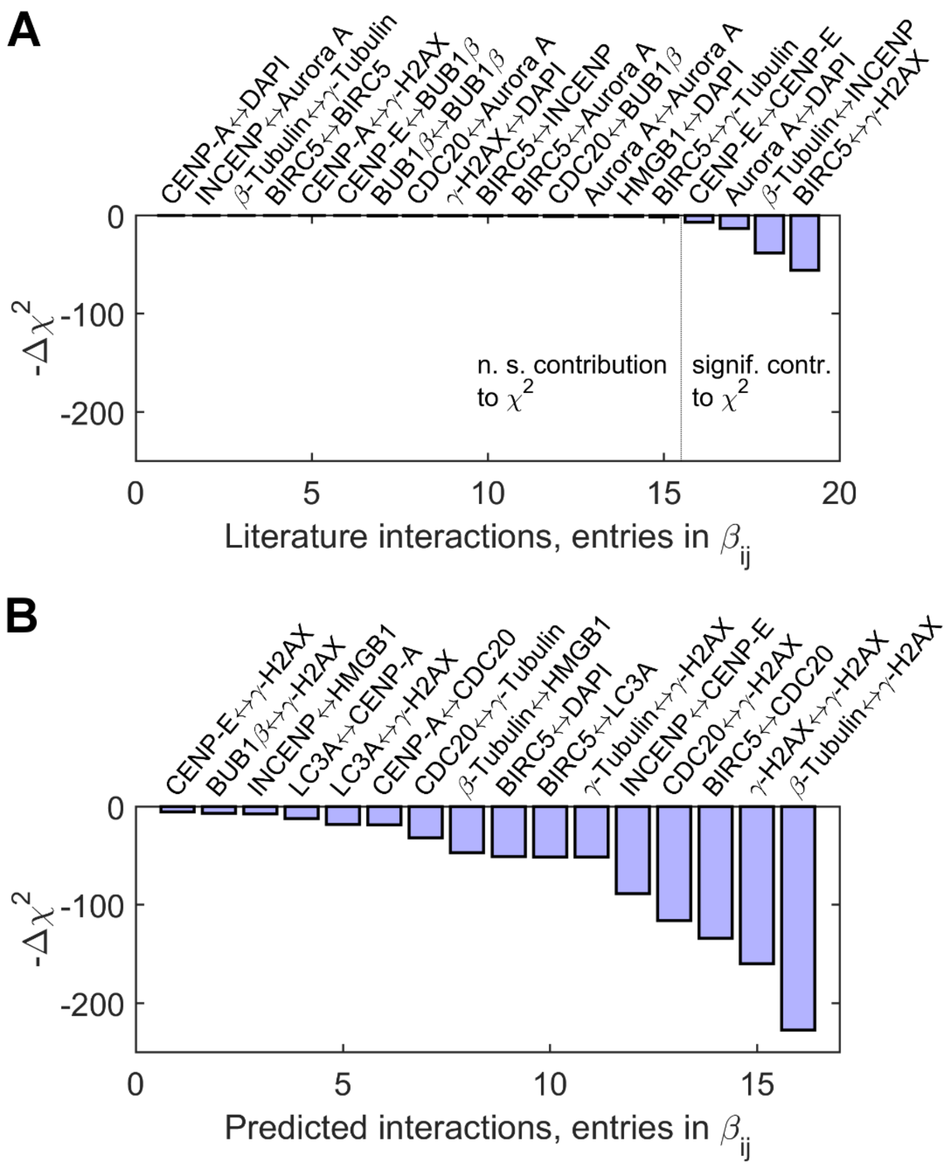

Appendix Figure S3 - Importance of affinity parameters for model fit.

A Affinity parameters related to literature interactions were withdrawn before refitting the model. $\Delta\chi^2$ differences between the full model and reduced models are shown. Only four literature interactions significantly contributed to explaining the dataset, based on a 95% confidence interval of a one-dimensional χ^2 distribution. Of note, the observation that an affinity parameter was not required for the model to explain the data does not imply absence of binding between these species but likely results due to non-identifiability.

B $\Delta\chi^2$ differences between the full model and reduced models, in which predicted affinities were withdrawn before refitting the model.

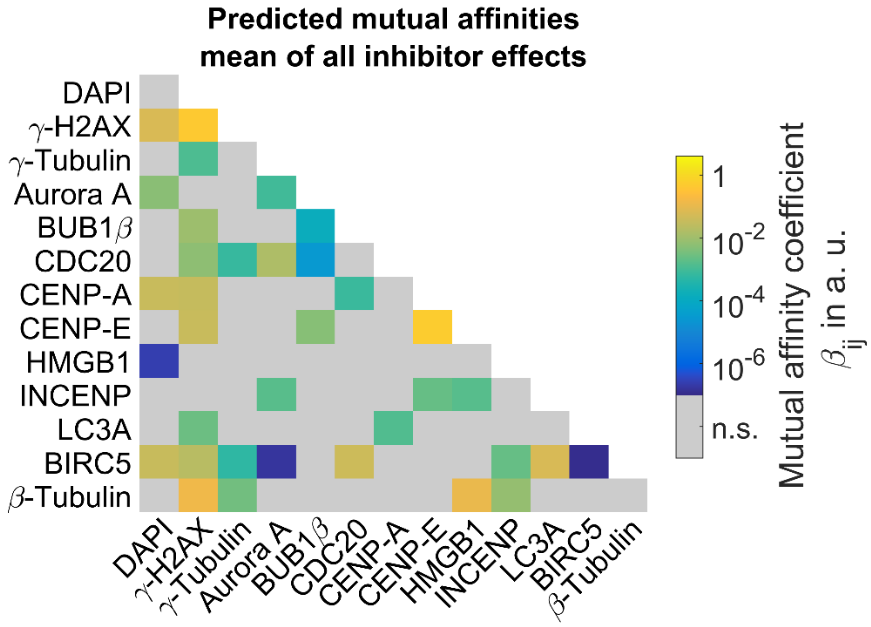

Appendix Figure S4 - Average mutual affinities of all inhibitor effects.

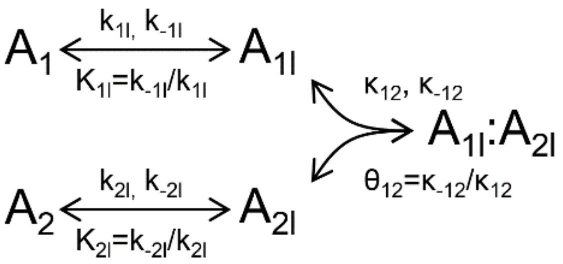

Appendix Figure S5 – Simple affinity model for two species.

Binding of species A_1 and A_2 to mitotic ROI l results in A_{1l} and A_{2l}. Mutual binding in this compartment results in A_{1l}:A_{2l}.

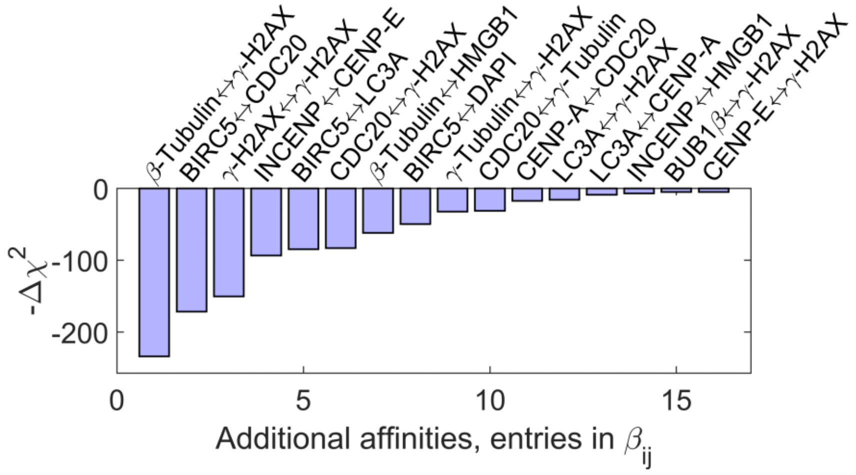

Appendix Figure S6 – Sequential forward selection of mutual affinities.

Affinities between proteins included in addition to literature interactions. By sequential forward selection, entries in the matrix β_{ij} that contains mutual affinities between proteins were included in the model if the model fit was significantly improved. All included affinities were ordered according to the improvement in the χ^2 measure of model deviation from the experimental data.

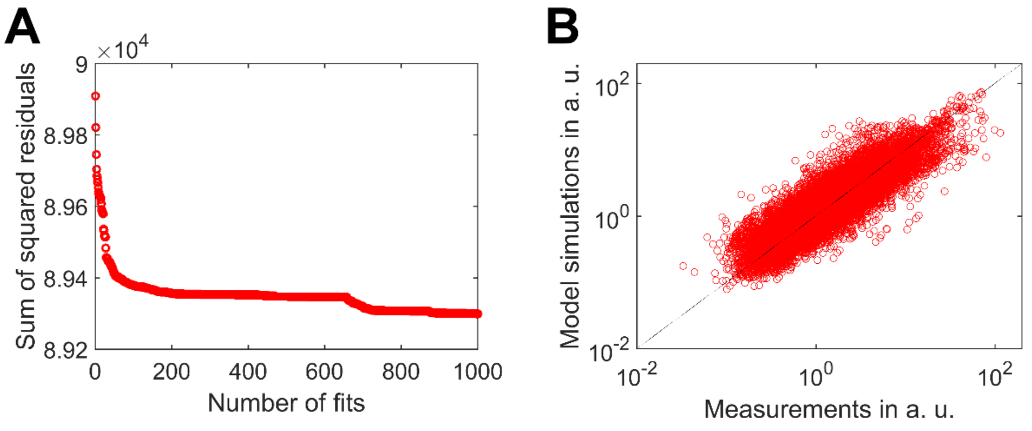

Appendix Figure S7 - Results of multi-start local optimizations.

A Ordered sums of squared residuals for 1000 model fits to the control dataset from untreated cells. Differences between best fits were below the range of squared residuals for single data points indicating convergence of model fits towards a global optimum.

B Best-fit model simulations and measurements indicate that the model is consistent with the experimental dataset.

Appendix Table S1 - Assay proteins, inhibitors and cell lines (A, antibodies and fluorophores; B, inhibitors and their targets; C, cell lines and their type).

A

**Antibody**	**Alternative name**	**Fluorophore**
Aurora kinase A	Aurora A	Cy3
BUB1β	BUBR1	DyLight 650
CDC20		DyLight 650
CENP-A		DyLight 650
CENP-E		DyLight 550
H2AX		DyLight 550
HMGB1		Cy3
INCENP		DyLight 650
MAP1LC3A	LC3A	DyLight 650
BIRC5	Survivin	Cy3
β-Tubulin		Cy3
γ-Tubulin		DyLight 550

B

**Target**	**Alternative name**	**Drug**
Aurora kinase A	Aurora A	MK-5108
Aurora kinase B	Aurora B	Barasertib
CENP-E		GSK923295
Chk1		MK-8776
Haspin		CHR-6494
KIFC1	HSET	CW069
Kif11	Eg5	Ispinesib
Microtubules		Paclitaxel
Microtubules		Vinblastine
PLK1		GSK461364
BIRC5	Survivin	YM155
Topoisomerase II		Etoposide

C

**Cell line**	**Type**
MCF10A	non-tumorigenic
MCF10CA	tumorigenic
