# Supplementary material for: Unraveling mitotic protein networks by 3D multiplexed epitope drug screening
Source: Mol Syst Biol. 2018 Aug 13;14(8):e8238. doi: 10.15252/msb.20188238 (PMC6088390; doi:10.15252/msb.20188238)
Supplement: Supplementary file 2 — Expanded View Figures PDF [file MSB-14-e8238-s002.pdf]

Expanded View Figures

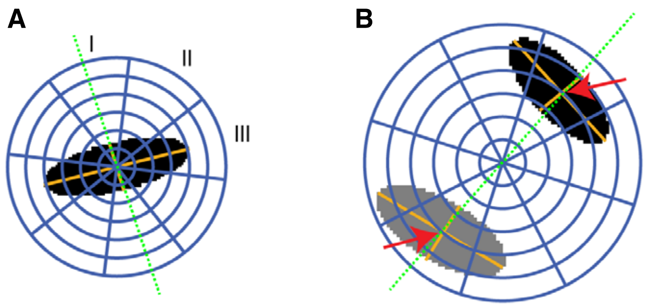

Figure EV1. Definition of spherical ROIs.

A In cells during metaphase, a 3D ellipsoid (black area) was fitted to the DAPI signal to determine the orientation of the mitotic axis (green dotted line). This axis was defined by the smallest axis of the ellipsoid. Relative to the mitotic axis, three spherical sectors (I, polar; II, diagonal; III, equatorial) were delineated. Six shells were defined by dividing the sphere radius into six equally large intervals, of which the inner four spanned the largest ellipsoid axis nucleus area. Finally, 18 spherical 3D partitions were defined as intersections between spherical sectors and shells.

B In cells during segregation, two ellipsoids were fitted to the chromatin regions (black and gray areas). The mitotic axis (green dotted line) was defined by the centroid to centroid vector. The map of spherical ROIs was specified relative to the centroid distances of the two chromatin regions. Six shells with equal radius intervals were defined by scaling the fourth shell to the distance between the centers of the two ellipsoids (red arrows).

 © 2018 The Authors

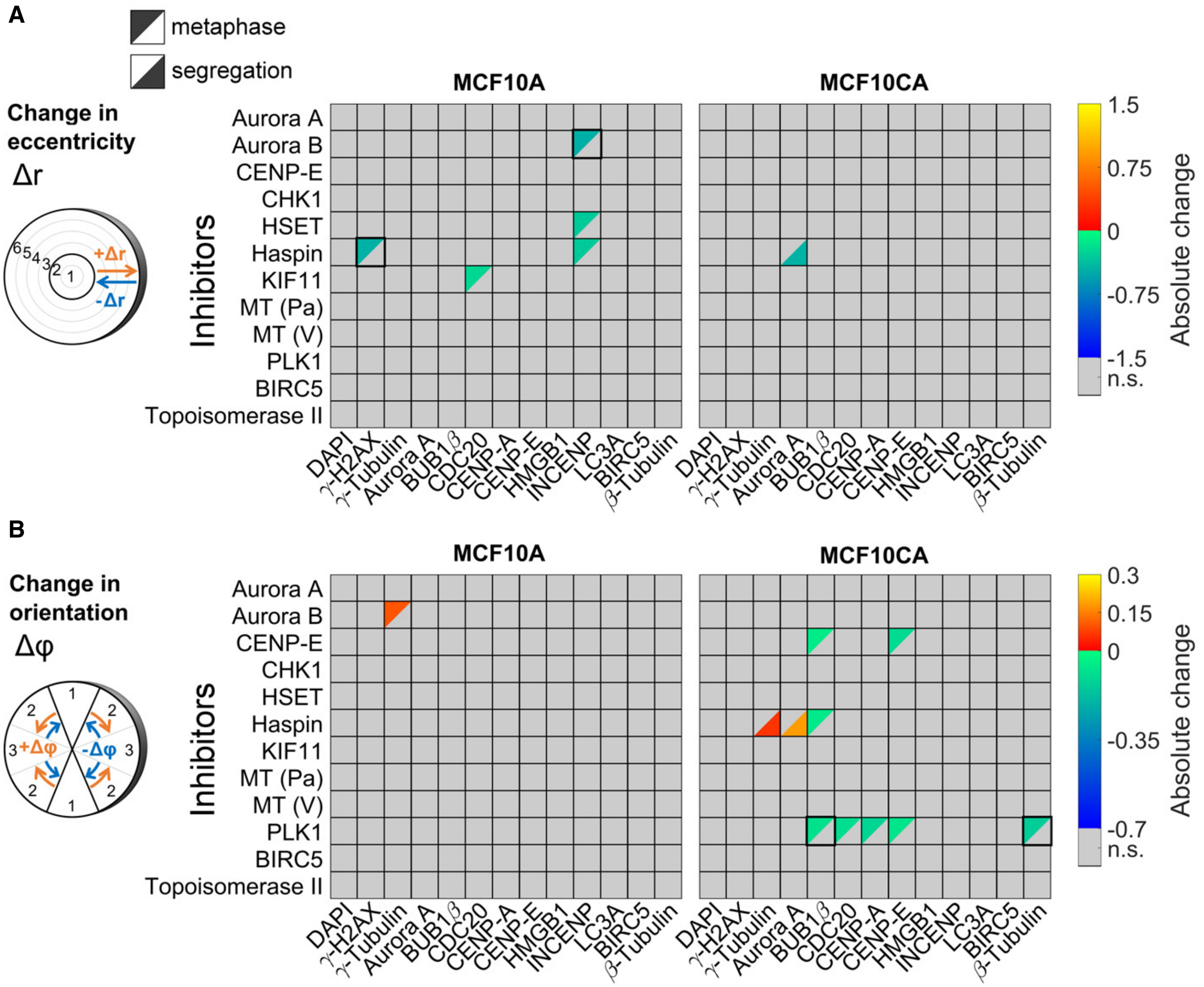

Figure EV2. Changes in eccentricity or orientation of distribution patterns due to inhibitor treatments.

A Changes in eccentricities of distribution patterns due to inhibitor treatments in MCF10A (left panel) or MCF10CA cells (right panel). All significant effects were visualized (Welch's t-tests performed for 18 ROIs followed by Bonferroni correction, $P < 0.05/18$). Positive values describe a movement to the periphery, while negative values represent a movement to the center of the cell (MT, microtubule inhibitor; Pa, paclitaxel; V, vinblastine; n.s., not significant).

B Changes in orientation of distribution patterns due to inhibitor treatments in MCF10A (left panel) or MCF10CA cells (right panel). All significant effects were visualized as in (A). Positive values describe a movement toward the plane perpendicular to the cell division axis, while negative values describe a movement toward the cell division axis.

Data information: Black frames indicate examples shown as SpheriCell plot in Fig 3C.

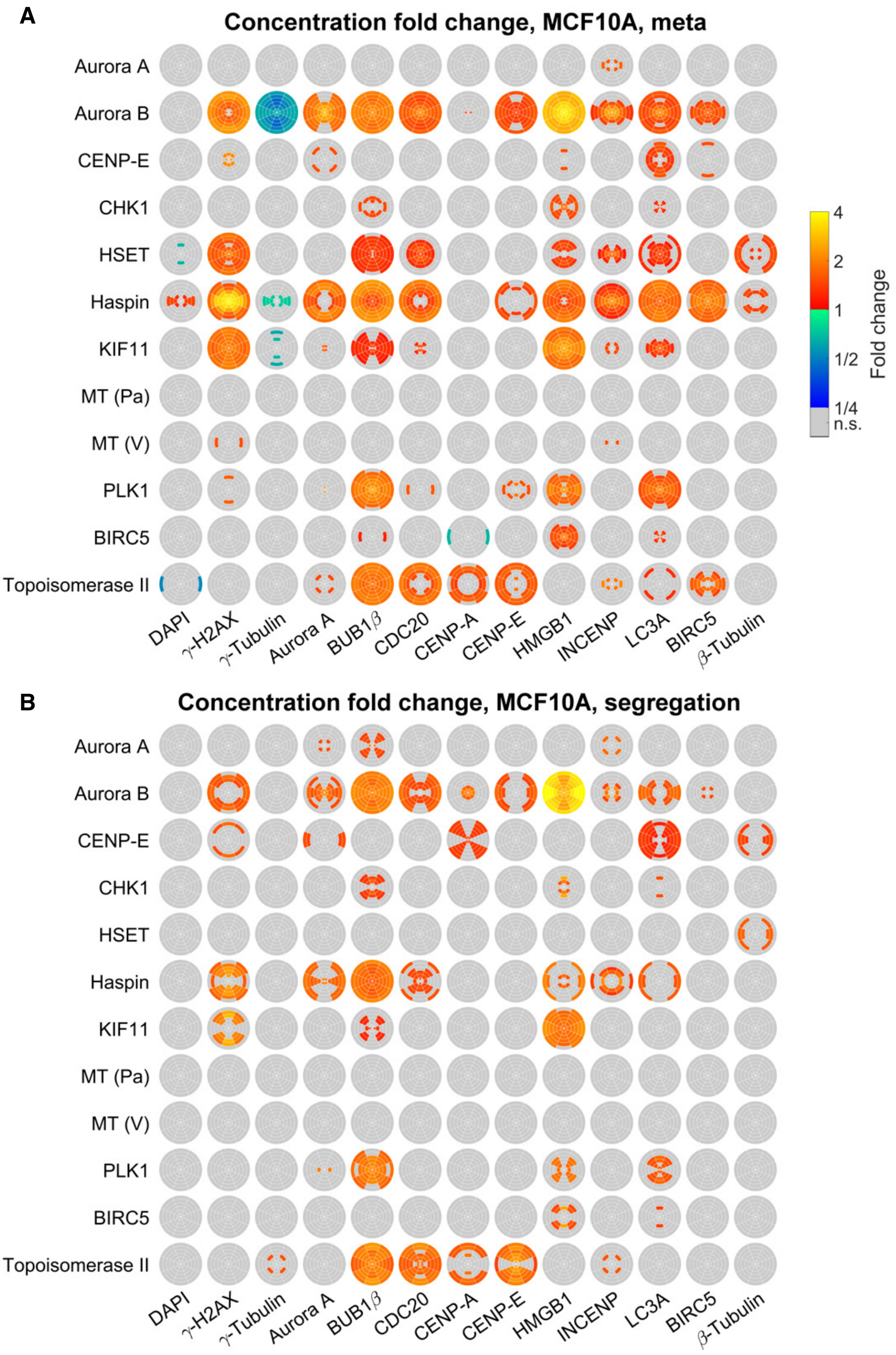

Figure EV3. SpheriCell plots for inhibitor effects in MCF10A cells.

A, B SpheriCell plots indicating concentration fold changes for measured species in metaphase (A) or segregation phase (B) MCF10A cells treated by 12 inhibitors. Effects were visualized in ROIs with significant effects (Welch's *t*-tests performed for 18 ROIs followed by Bonferroni correction, $P < 0.05/18$).

© 2018 The Authors

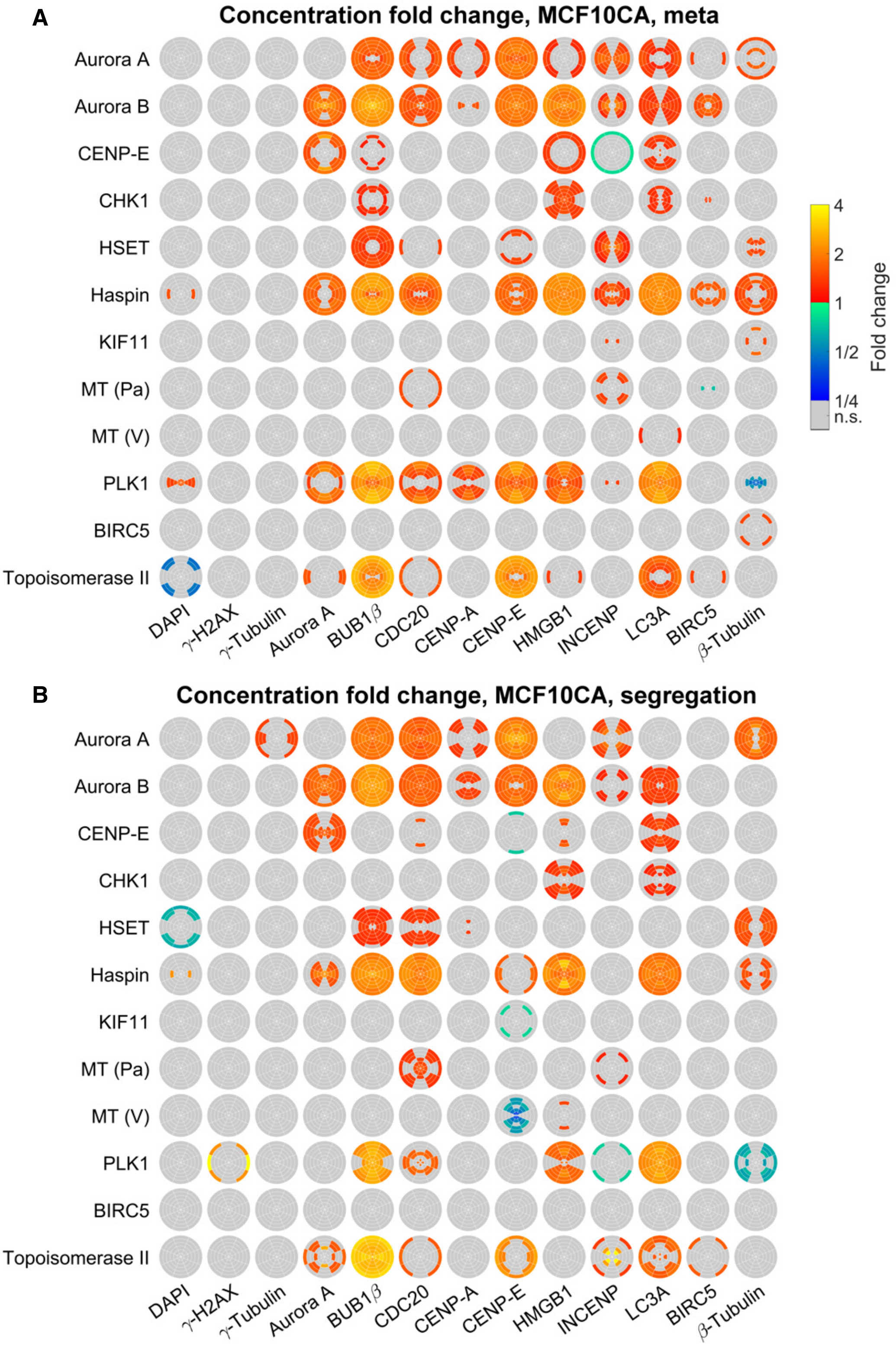

Figure EV4. SpheriCell plots for inhibitor effects in MCF10CA cells.

A, B SpheriCell plots indicating concentration fold changes for measured species in metaphase (A) or segregation phase (B) MCF10CA cells treated by 12 inhibitors. Effects were visualized in ROIs with significant effects (Welch's *t*-tests performed for 18 ROIs followed by Bonferroni correction, $P < 0.05/18$).

© 2018 The Authors
